# Social Ecology of Children’s Vulnerability to Environmental Pollutants

**DOI:** 10.1289/ehp.9101

**Published:** 2006-05-10

**Authors:** Bernard Weiss, David C. Bellinger

**Affiliations:** 1 Department of Environmental Medicine, Environmental Health Sciences Center, and Center for Reproductive Epidemiology, University of Rochester School of Medicine and Dentistry, Rochester, New York, USA; 2 Children’s Hospital Boston, Harvard Medical School and Harvard School of Public Health, Boston, Massachusetts, USA

**Keywords:** covariates, effect modification, environment deprivation, environmental enrichment, risk assessment, stress

## Abstract

**Background:**

The outcomes of exposure to neurotoxic chemicals early in life depend on the properties of both the chemical and the host’s environment. When our questions focus on the toxicant, the environmental properties tend to be regarded as marginal and designated as covariates or confounders. Such approaches blur the reality of how the early environment establishes enduring biologic substrates.

**Objectives:**

In this commentary, we describe another perspective, based on decades of biopsychological research on animals, that shows how the early, even prenatal, environment creates permanent changes in brain structure and chemistry and behavior. Aspects of the early environment—encompassing enrichment, deprivation, and maternal and neonatal stress—all help determine the functional responses later in life that derive from the biologic substrate imparted by that environment. Their effects then become biologically embedded. Human data, particularly those connected to economically disadvantaged populations, yield equivalent conclusions.

**Discussion:**

In this commentary, we argue that treating such environmental conditions as confounders is equivalent to defining genetic differences as confounders, a tactic that laboratory research, such as that based on transgenic manipulations, clearly rejects. The implications extend from laboratory experiments that, implicitly, assume that the early environment can be standardized to risk assessments based on epidemiologic investigations.

**Conclusions:**

The biologic properties implanted by the early social environment should be regarded as crucial elements of the translation from laboratory research to human health and, in fact, should be incorporated into human health research. The methods for doing so are not clearly defined and present many challenges to investigators.

In this commentary, we argue that the outcomes of exposure to neurotoxic chemicals early in life are shaped by the nature of a child’s social environment, including that prevailing before birth. Our guiding thesis is that toxicity is not simply an inherent property of the toxicant but derives from an assortment of jointly acting variables bound implacably into the individual. Neurotoxicology accepts genetic predispositions as intrinsic influences, but aspects of the broader environment, especially its social characteristics, tend to be either regarded, at best, as a marginal influence or dismissed as merely a nuisance, contributing a source of confounding bias. We contend that a true evaluation of toxic potential and its neurobehavioral consequences is inseparable from the ecologic setting in which they act and which creates unique, enduring individual vulnerabilities that warrant the same status as genetic predispositions and are imprinted as forcefully. Although aspects of this perspective are well accepted among neurotoxicologists, it has not yet found wide application in study design and analysis. Therefore, we also suggest factors, which are primarily methodologic, that appear to be important impediments.

## The Customary Approach to Human Studies

Environmental chemicals comprise only one of many exogenous factors that can influence child development. Recognizing this principle, investigators attempting to determine whether and how a particular agent induces developmental neurotoxicity strive to separate its unique contribution from those of the larger, particularly social environment. Such investigations tend to strip away those contributions (covariates, confounders) statistically through techniques such as multiple regression. The major goal of such analyses is to avoid misattributing to the neurotoxicant of interest an adverse effect that is actually due to one of these other factors. In such an analysis, a model of the outcome of interest is first constructed in which consideration is given only to the contributions of factors other than the neurotoxicant. In essence, the analyst tries to “explain” as much of the variation in the outcome as possible by reference only to these other factors. This effort never achieves complete success, leaving some of the variation “unexplained.”

The second step is to determine whether the neurotoxicant of interest accounts for a significant portion of this residual variance. Although such a strategy will fulfill its principal aim of reducing the risk of type I error, it carries the attendant risks of other types of inferential errors. The strategy is rather conservative: It values avoidance of mistaking what is a true social ecologic effect interwoven with a neurotoxicant more highly than the converse, which would represent a type II error. Such a strategy presumes that the influence of the selected social ecologic factors on the measured outcome is completely independent of the effects of the neurotoxicant. It further assumes that the potency of the neurotoxicant is invariant regardless of the social ecology within which it occurs. Such a bold assumption is appealing in its simplicity but almost certainly wrong. It steers us, in fact, to a scientific impasse. All “main effects” are misnamed because such entities are not readily identifiable. Each is the result of the factor of interest operating on the end point of interest in a particular ecologic context. Pearce (1989) summarized the problem by observing that “every effect estimate . . . reflects numerous unknown or unmeasured modifying factors.” A “main effect,” so to speak, merely describes an interaction that is incompletely characterized.

Although the current approaches may be convenient for segregating the role of a particular agent, they are less useful as a policy guide. Neurotoxicant exposures are not distributed randomly. They are chained to a multitude of other risk factors that resist partitioning. The focus of arguments about the relationship of developmental lead exposure to indices such as IQ, for example, is the ability of such studies to separate the contribution of lead itself from those of maternal IQ, family income and social class, marital status, prenatal care, maternal drug use, family caregiving, and other aspects of the child’s total environment. It is a puzzle reminiscent of the one posed by chemical mixtures, but, for even this simpler puzzle, solutions to questions of additivity, antagonism, and synergism continue to elude us.

From another vantage point, these presumably external factors represent not simply confounders, but what in epidemiology are called effect modifiers. That is, “[e]ffect modification . . . is present when the magnitude of the association between an exposure and an outcome varies across strata of some other factor” (Bellinger 2000). For example, the magnitude of the adverse effect of lead exposure on a measure such as the Mental Development Index of the Bayley Scales of Infant Development depends partly on the child’s social class (usually defined by parents’ occupational and educational levels). Unlike children from “lower-class” backgrounds, an adverse effect could not be demonstrated in children from “upper-class” backgrounds unless cord blood levels at birth exceeded 10 μg/dL. Winneke and Kraemer (1984) reported a similar finding. Analogous relationships were traced in assessments of how polychlorinated biphenyls (PCBs) affect neurobehavioral development, again demonstrating that nature is much more complex than our usual assumptions. In a study conducted in the Netherlands, prenatal exposure had adverse effects only among children with “less optimal” parental and home characteristics (Vreugdenhil et al. 2002). In a New York City (USA) cohort, Rauh et al. (2004) observed a significant interaction between prenatal residential exposure to environmental tobacco smoke (ETS) and socioeconomic disadvantage. The greatest cognitive deficits on the Bayley Scales emerged among children who both were exposed to ETS and had families that suffered material hardships, operationally defined as “unmet basic needs in the areas of food, housing, and clothing” (Rauh et al. 2004).

An important implication of these examples for risk assessment is that the “main effects” of lead or PCBs or ETS, estimated by multiple regression, are misleading characterizations of their respective associations with neurodevelopment. In effect, such measures represent weighted averages of the associations, summing across strata in which the association with neurodevelopment is stronger and strata in which it is weaker. Exposure standards that rest on such estimates will be more protective for the subgroup of children whose social ecology has rendered them relatively insensitive to the adverse effects of neurotoxicant exposure, but less, perhaps even insufficiently, protective for the subgroup of children whose social ecology has rendered them more sensitive.

## The Early Environment as a Variable

Much like the epidemiologic studies referred to above that attempt to hold constant, by means of statistical control, variables other than the specific exposure under investigation, animal experimenters often assume that typical laboratory conditions, because they seem free of the many complexities that plague human research, offer an environment in which the “pure” effects of a developmental neurotoxicant can be observed. The methods sections of such reports typically describe the housing and rearing conditions without comment—for example, by noting that the animals were maintained in “standard” cages or fed “standard” rodent diets. For another area of developmental neuroscience, such a lack of detail might be viewed with astonishment. This area is devoted to research on the neuro-behavioral consequences of environmental enrichment and deprivation. In the following section, we briefly summarize some key studies on the effects of environmental enrichment, postnatal stress, and prenatal stress. This literature, as observed by Laughlin (2000), provides valuable lessons for neurotoxicology.

### Environmental enrichment

The 1960s provided the earliest experimental evidence that modifying the environments of young rats, especially by adding complexity or enrichment, could alter the neurochemical and morphologic characteristics of their brains as well as their behavior (e.g., Bennett et al. 1964; Diamond et al. 1972 Diamond et al. 1974). The enriched environment consisted of a large cage with 10–12 animals and novel stimulus objects such as ladders, wheels, and blocks. The standard environment consisted of a conventional laboratory cage containing three rats. The rats raised in the enriched environment showed increased cortical thickness, increased sizes of neuronal cell bodies and synaptic contact areas, increased numbers and extent and branching of dendrites, and more synapses per neuron than did the rats raised in the standard environment (Diamond 2001; Rosenzweig and Bennett 1996). Later, it was shown that the effects of enriched and impoverished experience could be induced at almost any part of the life span, even with relatively short periods of exposure. The investigators found, for example, that they could obtain similar effects by assigning 50-day-old rats to enrichment for 30 days. Also, 2 hr/day in the differential environments for 30–54 days produced cerebral effects similar to those induced by 24-hr exposure. More recent research has confirmed these findings (Foster and Dumas 2001; Rampon et al. 2000a, 2000b) and extended them to end points such as brain growth factors (Ickes et al. 2000), neurotransmitter function (Naka et al. 2005), motor coordination, spatial discriminations, and other behavioral indices (Pham et al. 1999; Schrijver et al. 2002).

Substantial supporting evidence exists for the same phenomenon in humans. For example, studies of children raised in impoverished environments revealed cognitive deficits of substantial magnitude by 18 months of age (see Sameroff 1993).

### Postnatal stress

Another pertinent literature focuses on environmental effects on endocrine function, particularly the stress hormones (glucocorticoids), the hypothalamic–pituitary–adrenal (HPA) axis, and adult responses to stress. Until the experimental data emerged, responses to stress were deemed to be “innate” and not modifiable by experience. In rodent species, critical elements of brain development take place during the first 2 weeks or so of life: neuronal proliferation, dendritic branching, synaptogenesis, and the wiring of neural circuitry. During this period, environmental conditions play a major role in such processes. The most influential is the nature of maternal care because the infant rodent, an altricial species, is totally dependent on the dam for survival. When maternal care is modified by experimental manipulations, the effects on infant brain development and behavior occur across a broad range of measures and are detectable during adulthood.

Stress in this context is not unambiguously defined because different degrees of what are considered stressful manipulations may be qualitatively dissimilar in effect. Mild stress during the first week of life, imposed by maneuvers such as brief periods, perhaps 3–10 min in length, of handling the infant (e.g., stroking it) after withdrawal from the nest, is able to diminish the behavioral and hormonal responses to stress (of a different kind) during adulthood. Adrenal activity in response to stress conditions such as confinement is reduced, and more activity occurs in the open field situation. Such stress also results in improved performance in a radial maze, improved shuttle box avoidance acquisition, improved water maze performance, reduced evidence of anxiety on an elevated plus-maze, and diminished hippocampal neuronal loss and cognitive decline with age. Many experimenters have noted that handled pups elicit more licking and grooming from the dams and that the dams show a higher incidence of the arched-back nursing position. Increased maternal attention seems to be one of the sources of the positive outcomes.

In contrast, more severe types of stress during infancy produce distinctly different outcomes than does mild stress. For example, Matthews and Robbins (2003) removed litters of rat pups from their nest cages for 6 hr on 10 occasions between postnatal day (PND)5 and PND20. Later in life, the pups who experienced maternal separation showed markedly reduced behavioral responses to both primary and conditioned reward stimuli, elevated locomotor activity in anticipation of presentation of a daily food ration, a Pavlovian conditioned response, and an attenuated response to the activity enhancement effects of *d*-amphetamine. Low-dose cocaine self-administration was attenuated whereas high-dose self-administration was enhanced. The authors interpreted these findings as evidence of reduced central dopaminergic function. Others have also observed altered neurotransmitter function in other systems—for example, diminished GABA_A_ (gamma-aminobutyric acid) receptor binding during adulthood (e.g., Fries et al. 2004). Most critically, these widespread neurobehavioral alterations endure and are not attenuated with age. Fish et al. (2004) observed that variations in maternal care in rats induce variations in gene expression in offspring brains that, in essence, result in differing phenotypes (Weaver et al. 2004; Zhang et al. 2004). Cameron et al. (2005), reviewing the outcomes of poverty during early development, write that such effects on gene expression “represent, in part, the process by which variations in socioeconomic status (SES) are ‘biologically embedded.’” Seen from this perspective, the social ecology prevailing during early development, like genetic endowment, creates a unique biologic heritage.

### Prenatal stress

Postnatal stress is directed at the neonates. Another segment of the literature has explored relationships between stressful manipulations during pregnancy and their effects on the offspring. Operationally, stress manipulations have taken the form of daily restraint periods (e.g., confinement in a plastic tube), cold water immersion, inescapable electric shock to the tail, saline injections, and unpredictable noise. The literature provides compelling evidence that stress during gestation exerts widespread effects on neurobehavioral development that extend far beyond the period of infancy. Neurochemical effects include altered monoamine turnover (e.g., reduced dopamine turnover in striatum and nucleus accumbens) and increased dopamine turnover in prefrontal cortex (Newport et al. 2002). Morphologic changes include reduced cell proliferation in dentate gyrus and decreased neurogenesis in hippocampus evoked by a spatial learning task. Behavioral changes include altered locomotor activity patterns and enhanced sensitization to the locomotor effects of amphetamine. And, as with postnatal stress, prenatal stress enhances the response to stressful conditions in the offspring. For example, such offspring tend to spend less time in the open arms of the elevated-plus maze and exhibit retarded learning of both active and passive avoidance. Sexual differentiation of the brain and copulatory behavior are also altered by prenatal stress; male offspring may be feminized (Gerardin et al. 2005).

Early studies in humans of the effects of maternal stress focused on outcomes such as pregnancy complications, delivery complications, and birth weight. Subsequently, psychologists initiated research on the cognitive and emotional development of the child. Later studies have shown that stressful circumstances and events such as marital strife, loss of a spouse, and especially poverty are also associated with developmental delays or attention deficits in childhood and lifelong adverse health and neurobehavioral effects. Evans (2004) argued that even these circumstances fall far short of encompassing the multiple disadvantages faced by children living in poverty. He observed that “[a] limitation of psychological research on poverty is the absence of an ecological perspective” that extends beyond the family setting.

### Toxic interactions (lead, ethanol) with early environments

As environmental health researchers, our primary interest in this literature arises from its neurotoxicologic implications. To what degree do early environmental conditions determine the consequences of neurotoxic exposures? Are their effects sufficiently profound that ignoring them distorts our conclusions?

Lead is the environmental neurotoxicant whose entanglement with the social environment, as measured by SES, is clearest (Bellinger and Matthews 1998). Two studies have now shown that environmental enrichment can attenuate its developmental neurotoxicity. Schneider et al. (2001) exposed rats, beginning on PND25, to ordinary tap water or water containing 0.2% lead acetate. The rats had been assigned to two different environmental conditions: single-cage housing (isolation) or group housing (eight per group) containing a variety of stimulus objects. Lead-exposed animals raised in isolation tended to show impaired water maze performance compared with the other rats in which enrichment generally overcame the lead and isolation effects. The blood lead values differed between the groups—26 μg/dL for the enriched group and 34 μg/dL for the isolated group—a surprising outcome, but differences in drinking water intake were not measured and could have been determined by the housing situation.

Guilarte et al. (2003) adopted the approach, more relevant to humans, of treating female rats from before breeding until weaning. Lead acetate (1,500 ppm) was added to their diets during this period. At weaning, male litter-mates were assigned either to isolation housing (one rat per cage) or to an enriched condition (eight rats per cage containing a variety of stimulus objects). Water maze performance during adulthood served as a measure of spatial learning. Isolation combined with developmental lead exposure produced the poorest performance. Enrichment improved performance in lead-exposed rats to levels seen in the control and enriched-control groups. In addition, this study suggested some possible biologic bases for the behavioral differences. Not only was performance enhanced by enriching the environments of the lead-exposed rats, but brain chemistry was altered as well. Lead-associated deficits in *n*-methyl d-aspartate receptor subunit 1 and brain-derived neurotrophic factor gene expression in the hippocampus were markedly attenuated in the enriched rats compared with the isolated rats.

These two studies only begin to suggest the power of the early postweaning environment to modify the consequences of exposure to developmental neurotoxicants. A much wider array of functional end points, beyond the crudeness of the water maze, deserves investigation. Methods that can trace and quantify the course of complex learning, such as schedule-controlled operant behavior, offer perhaps the most promise, especially because they can provide homologous situations in animals and humans (e.g., Chelonis et al. 2004; Paule et al. 1999).

Wallace et al. (2003) examined this issue from a different standpoint. By exposing rats prenatally to a known developmental neurotoxicant, methylazoxymethanol acetate (MAM), they blunted the increase in visual cortical thickness produced by being raised in an enriched environment. This effect on experience-dependent neural plasticity occurred at much lower exposure levels than did the overtly toxic effects of MAM seen under standard conditions, suggesting that impairments in neuronal plasticity may be a more sensitive index of neurotoxicant exposure than are the toxicant’s direct effects. One implication is that positive interventions in disadvantaged communities, such as expanded educational resources, may be counteracted by levels of pollution much smaller than would be detected by conventional assessments.

Three recent reports (Cory-Slechta et al. 2004; Virgolini et al. 2005, 2006) illustrate how prenatal stress can modify the response to lead exposure. They were based on the knowledge that low SES is itself a risk factor for adverse health consequences, including neuro-behavioral function, that in many ways parallel lead neurotoxicity. Proceeding from the thesis that many of the effects of low SES arise from stress responses attributable to the HPA axis and glucocorticoids, the investigators exposed female rats to lead via drinking water 2 months before scheduled breeding. Exposure continued through lactation until weaning at PND21. Maternal stress procedures, carried out on gestational days 16 and 17, consisted of placing the dams in restraint tubes three times on each of those days. Measurements in offspring of schedule-controlled operant behavior performance, neurotransmitter function, and corticosterone levels revealed a series of complex interactive effects of stress and lead and marked sex differences. The authors’ conclusions provide a template for the next stage of environmental health research: “Greater attention to the problems arising from interactions of risk factors must certainly be considered, given that such a scenario, in contrast to individual chemical exposures, constitutes the environmental reality” (Cory-Slechta 2006).

## Implications for Toxicologic Studies

The animal data highlight the principle that even relatively modest differences in the quality and properties of the early environment exert a powerful influence on later neuro-behavioral function. Their effects range from vulnerability to stress in later life to cognitive performance, to emotional predispositions, to enduring changes in neurotransmitter function and brain morphology. Even as superficially simple a variable as cage size can exert significant effects. Wurbel (2001) argues that the usual housing environment for laboratory rodents is so at variance with their natural habitats that we typically study subjects, even before we undertake experimental procedures, whose neurobehavioral capacities have been distorted. Given the accelerated investments in mouse genetics and behavior, experimenters need to be aware that the early environment, such as housing condition, can eliminate or even reverse behavioral differences ascribed to genetics (e.g., Crabbe 1999).

These implications have been overlooked in toxicology, not just in areas bearing on nervous-system function but also in areas superficially remote from them. One example was provided by Strange et al. (2000) in their review of work on tumor growth and housing conditions. In their studies, they divided mice into two groups at weaning (PND21). One was housed individually; the other was assigned to group housing. At 2–4 months of age, the mice were injected with mammary tumor cells and divided into additional groups. These manipulations produced marked differences in tumor growth among the various groups. The conventional cancer bioassay procedure takes no account of housing conditions, and standard cancer studies begin to expose animals at 8 weeks of age.

### Effect modifiers as risk factors

How should our practices in risk assessment and toxicology incorporate what we have learned about the profound ways in which the ecologic setting prevailing early in life exerts effects on later neurobehavioral function? Perhaps we should discard the tenet, guiding much contemporary research, that we need to simplify the world to produce an unequivocal result. This view, with its emphasis on independent main effects, does not acknowledge that most adverse effects reflect the joint contributions of multiple sources. Even SES, as noted above, is far from a unitary dimension; the label itself is an umbrella covering a multitude of variables (Bellinger 2000, 2001) that endure as biologic substrates (Fish et al. 2004).

Skepticism about conventional approaches is warranted in another way. The terms “effect modification,” “covariate,” and “confounder” imply external sources. This is not an accurate depiction. The reality is that there are no external sources. What often are labeled as effect modifiers or covariates are bound inextricably, in the individual, with the measures they are presumed to influence from the outside. Many, perhaps most, effect modifiers actually define different populations.

**Figure 1:**
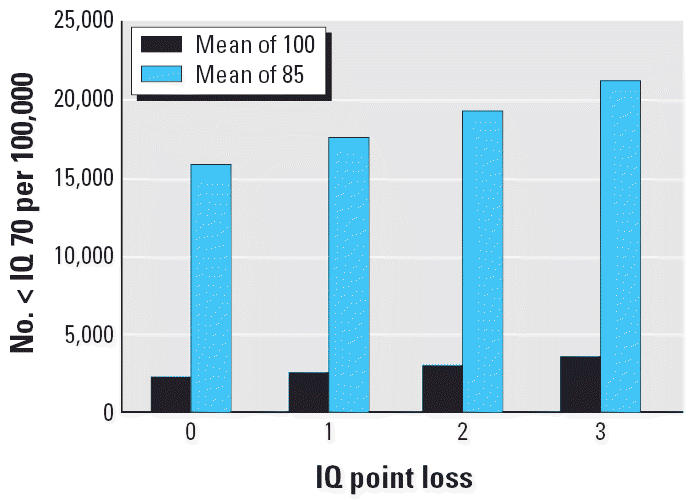
Advantaged communities typically show about a 15-point or 15% higher mean IQ score compared with disadvantaged communities. If both populations, as a result of neurotoxic exposure, suffer an equivalent decrease in mean IQ, the effect will be greater in the disadvantaged community, as gauged by the number of IQ scores < 70, although both populations suffer. Despite recognition that the IQ distribution is a continuous variable, a score < 70 is often taken as evidence of retardation and, in many school districts, requires remedial education (Weiss 2000).

Consider the situation depicted in Figure 1 (Weiss 2000), which compares the effects of shifts in mean IQ in two different communities, one that would be ranked as advantaged and one as disadvantaged. “Advantaged” and “disadvantaged” are terms that embody different suites of risks and different ecologic settings. Poverty in its many dimensions accounts for a large proportion of such risks.

In many surveys, the differences in mean IQ scores of such populations approximate 15 points (e.g., Sameroff et al. 1987) or about 1 SD on intelligence tests such as the Wechsler Preschool and Primary Scale of Intelligence. Assume, for modeling purposes, initial IQ distributions with respective means of 100 and 85, both with SDs of 15. As an impact index, calculate the number of scores < 70. Conventional education standards tend to assume that a score < 70 indicates the need for remedial measures and, for some school systems, signifies a classification as “retarded.”

With population sizes of 100,000 each, as shown in Figure 1, a loss of 1 IQ point (1%) in the advantaged population will increase the number of individuals < 70 from 2,280 to 2,660. In the disadvantaged population, the loss assigns 17,530 rather than 15,870 individuals to the < 70 category. Although the proportional shift is greater in the advantaged population (16.7%) than in the disadvantaged population (10.5%), the number of individuals added to the developmentally disabled category is much larger in the disadvantaged population (1,660) than in the advantaged population (380). The discrepancies enlarge with greater IQ losses, which could result from higher neurotoxicant exposures. This represents another aspect of the argument, also made by Bellinger (2000), that it is fruitless to search for a single point estimate for a “true” relationship between exposure and an end point such as IQ because the distributions of factors that affect the relationship will almost certainly differ in many respects between two populations. Insofar as each individual’s ecology is, at some level of detail, unique, the point estimate can be expected to differ for each individual. The group point estimate therefore represents the central tendency of the distribution of the estimates of group members.

**Figure 2:**
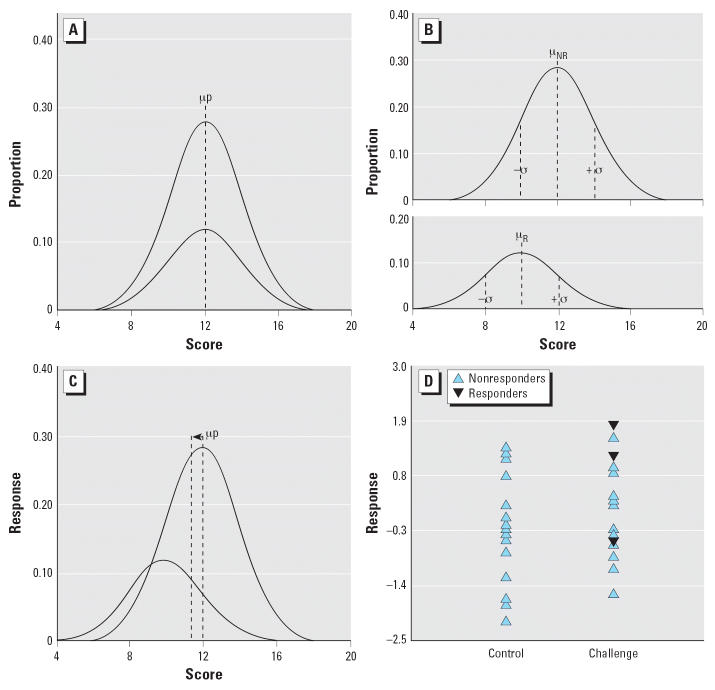
Influence of a susceptible subpopulation in a sample exposed to a toxic challenge. Abbreviations: μ, mean; NR, nonresponders; p, whole group; R, responders. (*A*) A hypothetical distribution generated to contain a sample composed of 30% responders (lower curve) and 70% nonresponders. (*B*) Distribution after exposure to a presumed neurotoxic agents that shifts the scores of the responders, on average, by 1 SD. (*C*) Because responders would not be identified beforehand in the usual study, the total distribution after challenge would display, as shown here, a negligible shift in the mean. Such a small shift would generally be taken as evidence of no effect, a conclusion that fails to assimilate the original hypothetical distribution. (*D*) A randomly chosen sample of 15 individuals from the total population after challenge was generated to show how, in such a sample, there is a marked overlap with control conditions despite the presence of a substantial proportion of responders.

The model in Figure 1 defined two different populations. Most investigators who knew beforehand about their disparities would not choose to combine them into a single population to study the effects of an environmental agent to which both are exposed. Most studies, however, do not seek to classify specific subgroups or to strive for homogeneity. Figure 2 depicts why heterogeneous samples can be misleading. Figure 2A illustrates a situation in which the sample population includes a sub-population (30%) inherently sensitive to the exposure of interest. In the absence of such a challenge, the sample population appears homogeneous. In response to the challenge, the mean of the sensitive subpopulation is shifted by 1 SD (Figure 2B). The entire sample, however, yields a distribution like that of Figure 2C, which exhibits, at most, only a slight shift in the mean. That slight, superficially negligible shift would be misleading because it ignores the reality of a major change, equivalent to 1.0 SD, in the sensitive subpopulation. Figure 2D provides one example of how a random sample of 15 subjects chosen from the total population might reflect exposure to a toxic challenge if the sensitive subjects responded with a shift of 1.0 SD.

**Figure 3:**
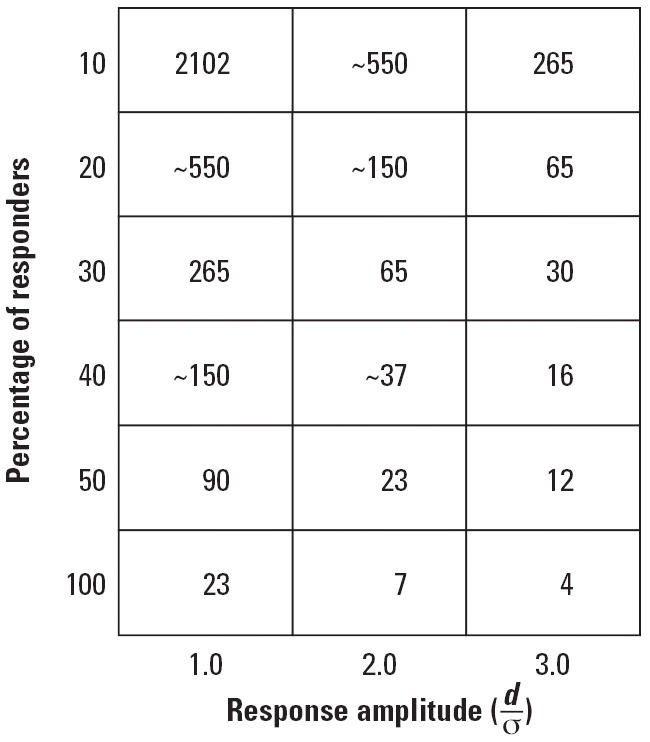
Sample size required to demonstrate a statistically significant effect (*p* = 0.01) 90% of the time for different magnitudes of response (effect size) and different proportions contributed by sensitive subpopulations. The ability to detect a small overall change when only a small proportion of the total population consists of responders requires extremely large sample sizes.

Figure 3 underscores the difficulties posed by such a confounding of subpopulations. It shows how large a sample would have to be enrolled to be able to detect displacements of a specified value if the subpopulation had not been identified in advance. In terms of Figure 1, if the disadvantaged population had been enrolled in a larger study in which it comprised 30% of the total sample, its response to a developmental toxicant would have been smothered in the analysis.

### Encompassing ecology

Although the likelihood that the social ecology of neurotoxicant exposure affects its expression is not an entirely novel concept, several factors appear to be impediments to fully addressing it in studies. Addressing these obstacles can be viewed as a research agenda. First, investigating effect modification requires that the factors most likely to modify neurotoxicity within a specific setting are known, incorporated into the study design, and measured well. Second, it is necessary that the joint distributions of the exposure of interest and the potential modifiers provide an adequate opportunity to assess a potential interaction. In general, assessments of effect modification require larger study cohorts than assessments of main effects. Because of the large number of potential effect modifiers, and the constraints that will be imposed by incorporating them into the design, the choice of which factors to focus on will necessarily be limited and will inevitably require trade-offs. Third, the multilevel modeling methods that are required to integrate individual-level, neighborhood-level, and community-level variables have not yet been widely applied in neurotoxicologic research. Expertise in the application of such analytic methods is much more common in the social than in toxicologic sciences. Fourth, our thesis that the “imprinting” of social-ecologic factors has its roots even in the prenatal environment implies that it is necessary to implement the costly and time-consuming longitudinal study designs that permit the systematic, prospective collection of high-quality data from that period.

Perhaps most important, to address the dependence of neurotoxicant effects on the social-ecologic environment in which exposure occurs will require strategies for measuring ecologic variables that are more robust and specific than those currently applied. (Of course, one can argue that this would be necessary even if the primary goal is to improve assessment of confounding bias attributable to aspects of the social ecology.) The social-environmental factors measured in neurotoxicity studies, whether subsequently modeled as potential confounders or effect modifiers, have generally consisted of individual- or family-level characteristics. Any influence on child neurodevelopment of neighborhood or community characteristics is assumed to be mediated entirely by such factors. But these more immediate factors are entrenched in a wider social and cultural setting integrated with the more traditional disease models that focus on biologic response as a function solely of host characteristics (Diez Roux 2004). The concepts and methods needed to assess such factors have been developed to a much greater extent by investigators in social epidemiology and in the broader social sciences. Collaborations with such investigators will become increasingly important.

Recent work in social epidemiology illustrates how the traditional approaches are unlikely to leave us with adequate models. For example, Rauh et al. (2003) found that even when individual-level risk factors for low reading scores were controlled in the analysis (e.g., males, low birth weight, low maternal education), community factors such as poverty indicators and percentage of immigrants remained significant predictors. Moreover, a cross-level effect emerged: A high percentage of immigrants in the community conferred a greater positive benefit on boys’ reading scores than on girls’ reading scores. This social-ecologic perspective must be incorporated in neurotoxicity studies if we are to implement “truly integrative, multilevel research strategies that consider the pathways to health operating at and between the social, cultural, individual, and biological levels” (Bachrach and Abeles 2004). The application of such strategies generates complex, multilevel data sets, but only in this way will our models begin to approach the true complexity of associations as they exist in nature.

Such issues and questions are not unique to neurobehavioral epidemiology. Environmental ecologists confront questions that in many ways are analogous. For example, in assessing metal toxicity, they have to take account of how different environmental conditions across the United States influence metal biogeochemistry rather than setting one universal standard. They may, for example, define a set of exposure scenarios and conduct comprehensive analyses for each. For a specific location, their assessments would include the chemical form of the metal as it enters the environment, the environmental conditions affecting its form and distribution (e.g., soil geochemistry, rainfall), and the effects that particular forms will have on target organisms. The parallels with health risk assessment are intriguing because each of these variables can be considered effect modifiers.

**Figure 4:**
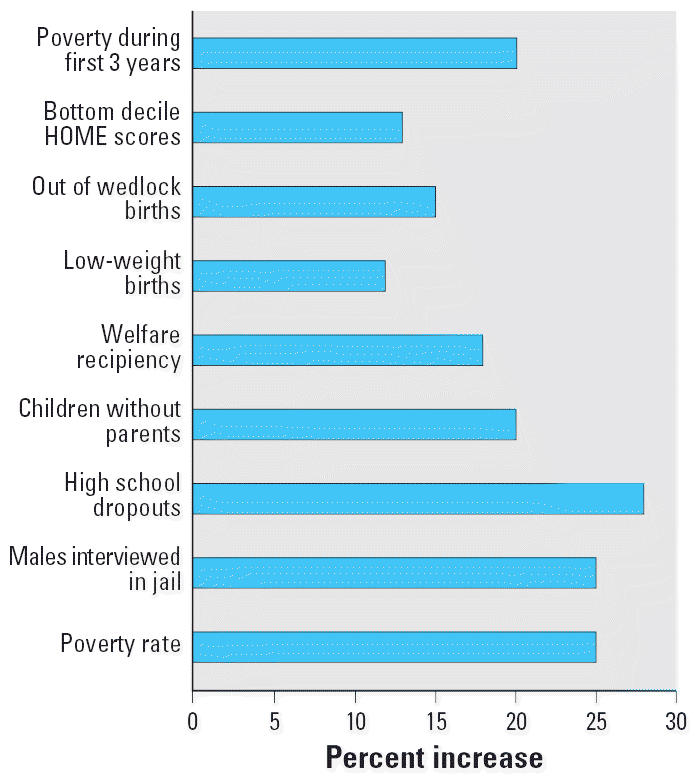
Estimated consequences of a 3% loss of IQ, based on calculations in Herrnstein and Murray (1994), showing percent changes in the selected indices. HOME, Home Observation for Measurement of the Environment (Caldwell and Bradley 1984).The calculations are based on the National Longitudinal Survey of Youth, a data set provided by the U.S. Bureau of Labor Statistics (Herrnstein and Murray 1994).

One perspective on the influence of the communal environment is plotted in Figure 4. Herrnstein and Murray argued in *The Bell Curve* 1994 that IQ, in essence, determines one’s status in society. They calculated the benefits to society of a 3% rise in IQ in their population. If the effects are symmetrical, as we have assumed in Figure 4, then a 3% lowering of IQ should produce an equivalent loss to society. But, we maintain, isn’t the argument inverted? Don’t the outcomes depicted in Figure 4 represent, instead, a constellation of social-ecologic variables contributing to or even determining the IQ loss? We certainly understood in 1994 the potency of the social environment in shaping child development. The *Bell Curve* argument is an unintended verification on an ecologic scale.

## Translations into Human Health

The National Institute of Environmental Health Sciences (NIEHS) 2006 strategic plan (NIEHS 2006) was formulated to promote “models for research that integrate patient-oriented or public health research with basic mechanistic studies to address disease etiology, pathogenesis, susceptibility and progression.” Such an objective will prove elusive if basic and mechanistic as well as human research fails to take into account the complexity of the human setting. Primarily, it must acknowledge that variations in the early environment create biologically distinctive organisms. Consider how toxicology has exploited the tools of contemporary molecular biology to create novel organisms such as mice with unique genetic properties. These genetically modified mice are tested for how they differ from ordinary mice in their response to toxic chemicals. Scientists carry out such a strategy because they know that toxicity is not a property of the chemical alone but, like the infectivity of a bacterium, a property shared jointly with the host. We deceive ourselves in the laboratory by assuming that testing animals raised under conventional conditions yields a “pure” evaluation of toxic potential free of the complexities that obscure human research. Like investigators trying by statistical surgery to isolate the effects of toxic chemicals on human development from its environmental setting, we cannot ignore the history of the host. The early environment, including that prevailing before birth, is not simply an influential variable but a biologic determinant. It creates qualitative rather than quantitative variation.

The core of our argument, then, is that the enduring biologic consequences of the individual’s developmental ecology are inseparable from how and to what extent he or she will be affected by neurotoxicant exposure. If genes are not treated as confounders but as biologic givens, neither should the vulnerabilities or protective mechanisms conferred by social ecology. Sociomics, so to speak, might, in fact, be an apt description of such a tenet.

The pioneering research of Cory-Slechta (2006) and others is forcing us to confront the reality that the association between a defined chemical exposure index and a defined neurodevelopmental outcome depends on a variety of host characteristics imprinted, so to speak, by the properties of the early environment. Current practices consolidate these properties into gross indices such as SES and, as well, tend to ignore the broader community setting. We know enough now to recognize that what we have labeled as covariates, confounders, and effect modifiers possess a biologic reality that we have yet to resolve. Solutions to these dilemmas do not unequivocally present themselves, but we hope to stir a colloquy among our colleagues.
